# Atypical lymphangioma and hyperkeratosis in a patient with morbid obesity^☆^^☆☆^

**DOI:** 10.1016/j.abd.2019.11.008

**Published:** 2020-05-05

**Authors:** Cristian Morán-Mariños, Wendy Nieto-Gutierrez, Josmel Pacheco-Mendoza

**Affiliations:** a Bibliometric Investigation Unit, Universidad San Ignacio de Loyola, Lima, Peru; b Institute for the Evaluation of Health Technologies and Investigation, Seguro Social de Salud del Perú, Lima, Peru

**Keywords:** Ichthyosis, Lymphangioma, Obesity, morbid

## Abstract

Lymphangioma is a rare and understudied pathology that is usually detected in the first decade of life, and its appearance in adults is rare. This report details a 51-year-old man with morbid obesity who presented, for the last eight months, multiple asymmetric tumor lesions with extension to the scrotal region. The diagnosis of circumscribed lymphangioma with associated infection was confirmed. This case report demonstrates an unusual presentation of the characteristics of the lymphangioma that are seldom described in the literature.

## Introduction

Circumscribed lymphangioma (LC) is infrequent and usually develops congenitally, being detected up to 90% of the cases in the first decade of life; however, it can also develop in adulthood.^1^

LC in the adult generally appears in areas affected by trauma, infection, or radiation, but it is also reported to appear spontaneously.^2^ It is described clinically as lumpy papules, and as verrucose or firm vesicles with a smooth surface, and is classified as a plaque, characterized by its lymphatic content. Histopathological examination describes dilated lymphatic vessels in the papillary dermis that elevate the epidermis above the surrounding skin.^2^

Reports of large-volume lymphangioma are very seldom described in the literature.

## Case report

A 51-year-old male from Peru, with a history of liver failure and morbid obesity, was transferred to the emergency department with a time of illness of eight months due to the presence of small tumor lesions at the pubic level, which evolved to become large.

**Figure 1:**
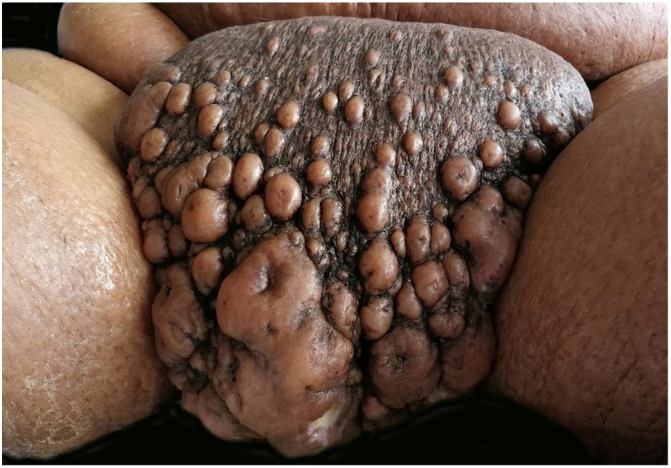
Multiple, grouped lesions of irregular size with purulent discharge.

Upon physical examination, multiple lesions with a cystic appearance, conglomerated, with raised and umbilicated borders were evidenced in the pubis; with a diameter of up to13 cm × 7 cm in which, through their orifices, they presented a purulent and foul exudate. The lesions extended to the scrotal region where they were associated with grouped vesicles that bled during mobilization (Fig. 1).

The lower limbs showed scaly and cracked plaques with hard-consistency hyperkeratosis due to chronic lymphangitis.

**Figure 2:**
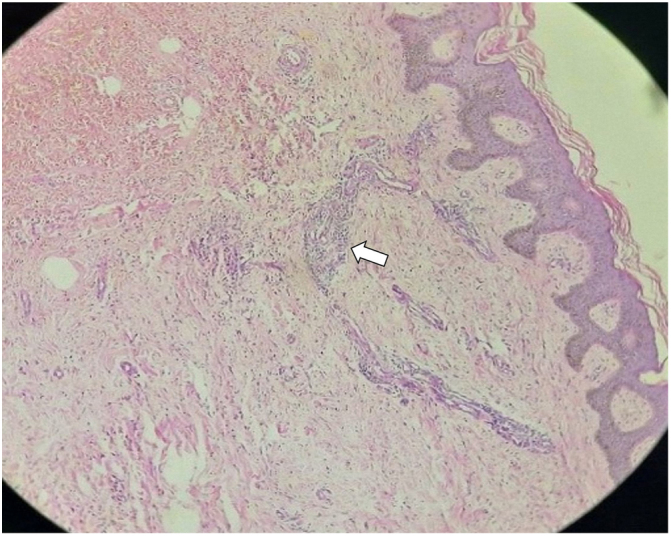
Proliferation of dilated lymphatic vessels with thin, irregular walls, lined by soft endothelial cells in the papillary dermis and with non-atypical lymphocytic aggregates; histopathology (Hematoxylin & eosin, x10).

Histopathological studies of the biopsy concluded with the diagnosis of circumscribed lymphangioma (Fig. 2). The patient was treated with daily cures and antibiotics such as oxacillin and clindamycin during his hospital stay. After two weeks of treatment, the patient progressed with favorable evolution of the lesions, with a decrease in size at the level of the pubis and scrotal region; however, due to the patient's underlying liver disease, he died a month before he could undergo surgery.

## Discussion

Lymphangioma is an infrequent and benign malformation of the lymphatic system, which can be classified according to the size or depth of the lesion. Although the authors report this entity as having a large volume, it lacks malignant potential, explaining the increase in size due to its ability to branch out and grow in a disorderly manner.^3^

The location of this entity is more frequent in the extremities, neck, and trunk, while the appearance in the scrotal region is rare.^4^ The lesions described in the patient are not those that are usually mentioned in the literature; the time of illness and the presence of associated infection, and morbid obesity, which probably contributed to the magnitude of the volume and expansion, must be considered. Moreover, a significant reduction of the size and secretion of the lesions was observed due to the use of antibiotics and daily cures.

The examiner should consider the characteristics of the lesions, which may be confused with condyloma acuminatum, molluscum, contagiosum, or herpes. A thorough inspection and an adequate clinical history can exclude these differential diagnoses.^5^

In lymphangiomas, the basic treatment without risk of infection involves the use of surgical resection, CO_2_ laser, or sclerotherapy; however there is the possibility of a spontaneous regression after three months that happens only in the macrocystic lymphangioma, while in the rest, the risk of recurrence appears between six months to six years.^6^

The biopsy sample was considered to be a limitation because it was not representative, since some histopathological characteristics were not similar to those reported by other studies.^5^ When the sampling was to be repeated in the scrotal region, the patient had died.

In summary, lymphangioma is an infrequent pathology in adults and the authors infer that the time of evolution, infection, and morbid obesity of the patient could have contributed to the atypical clinical manifestations described.

## Financial support

None declared.

## Authors’ contributions

Cristian Morán-Mariños: Statistical analysis; approval of final version of the manuscript; drafting and editing of the manuscript; collection, analysis, and interpretation of data; intellectual participation in the propaedeutic and/or therapeutic conduct of the studied cases; critical review of the literature; critical review of the manuscript.

Wendy Nieto-Gutierrez: Approval of final version of the manuscript; drafting and editing of the manuscript; critical review of the literature; critical review of the manuscript.

Josmel Pacheco-Mendoza: Approval of final version of the manuscript; drafting and editing of the manuscript; critical review of the literature; critical review of the manuscript.

## Conflicts of interest

None declared.
